# Planning and layout of tourism and leisure facilities based on POI big data and machine learning

**DOI:** 10.1371/journal.pone.0298056

**Published:** 2025-03-04

**Authors:** Shifeng Wu, Jiangyun Wang, Yinuo Jia, Jintian Yang, Jixiu Li

**Affiliations:** 1 School of Tourism, Hebei University of Economics and Business, Shijiazhuang, China; 2 Beijing-Tianjin-Hebei Sports Economy Research Base, Hebei University of Economics and Business, Shijiazhuang, China; 3 School of Hospitality and Tourism Management, University of Surrey, Stag Hill Campus, UK; 4 Hebei University of Economics and Business, Shijiazhuang, China; UCL: University College London, UNITED KINGDOM OF GREAT BRITAIN AND NORTHERN IRELAND

## Abstract

The spatial arrangement of tourism cities and the strategic placement of tourism and leisure facilities are pivotal to the development of smart tourism cities. The integration of Point of Interest (POI) data, enriched with location-specific insights, holds significant potential for urban planning and the optimization of spatial layouts. This study employs machine learning methodologies to evaluate the suitability of Beijing’s main urban area for the introduction of new tourism and leisure facilities. Drawing on POI and demographic data, and considering the distribution patterns of existing tourism and leisure facilities, this research applies machine learning to quantitatively simulate the optimal siting of such amenities. Key findings include: Firstly, compared with the existing tourism and leisure facilities, the fitting degree tested by the machine learning algorithm is 83.5%, suggests that the proposed method is highly feasible. Secondly, the decision-making model, trained with the CART algorithm, reveals that accommodation availability, shopping choices, and transportation infrastructure significantly influence the siting of tourism and leisure facilities in Beijing’s urban core. Thirdly, the model training indicates that facilities at various levels in Beijing exhibit a centralized layout, aligned with the city’s central axis, with a higher concentration in the urban center than in peripheral regions. The predictive analysis suggests that new tourism and leisure facilities are likely to be concentrated in densely populated areas. Lastly, some areas currently devoid of tourism and leisure facilities are identified as prospective sites for development. It is recommended that these areas be prioritized for the strategic placement. By leveraging machine learning algorithms for facility siting, this study aims to enhance the overall urban layout while mitigating the inherent subjectivity in planning and location decisions, offering valuable insights for the site selection of diverse facilities.

## Introduction

Urban tourism and leisure facility planning and layout constitute a pivotal domain within tourism research. Such studies are instrumental in both the formulation and refinement of urban planning, as well as in the optimization and establishment of urban service economy. The rapid development of tourism in recent years has played an increasingly important role in the economic landscape of Beijing. The study on the planning and layout of tourism facilities in the main urban area of Beijing is not only the key to improve the efficiency of tourism development in Beijing, but can also enhance the image of the city, and improve the happiness of urban residents and the experience of tourists [1]. Site selection is a critical and practical component of urban leisure tourism spatial planning. Traditional data acquisition methods, such as field investigation [2] and in-depth interview [3], have been the basics of this process. However, these methods present several limitations. Firstly, evaluations are often conducted using score cards, which results in scattered, limited, and homogeneous data. Secondly, the decision-making process is prone to subjectivity and sometimes lacking objective explanations. Thirdly, manual data collection is inefficient, inaccurate, and costly [4]. The advent of the big data era has introduced new methodologies that transcend the constraints of traditional human knowledge, time, and energy. Digital footprint [5], mobile positioning data [6], geotagged photos [7] and POI data offer a wealth of information that can inform more scientific and rational decision-making. These sources significantly reduce the costs and improve the efficiency of data collection [8–10]. POI data, in particular, is highly valued in urban functional space research due to its accessibility, extensive volume, and precise geolocation capabilities. It enables the creation of diverse alternatives based on data-driven insights, facilitating more informed decision-making in urban tourism planning [11,12].

This paper leverages machine learning techniques to conduct a detailed spatial analysis of the main urban area of Beijing. Utilizing a 1-kilometer grid scale, the study integrates Point of Interest (POI) data and population data, while also considering the distribution patterns of existing urban tourism and leisure facilities [13]. Initially, the study identified 629 potential sites for tourism and leisure facility construction from a total of 1548 grids within the urban area. Subsequently, by evaluating areas based on population density, 240 sites were selected for prior development. This approach aims to quantitatively simulate the optimal location layout for tourism and leisure facilities and to achieve a fine-scale, data-driven site selection process.

## Layout of tourism and leisure facilities

### Planning and layout theory of tourism and leisure facilities

Tourism and leisure facilities are integral to the fabric of urban tourism, serving as both foundational elements for tourism activities and services, as well as attractions. Cities act as the primary platform for tourism development, with urban leisure tourism drawing upon the city’s unique resources and advantages to flourish [14]. Regardless of the importance, a review of the literature on urban facility planning and layout reveals several research gaps. Firstly, existing studies tend to concentrate on specific types of facilities, such as sports or pension facilities, with public sports facility layout planning being a prevalent example. However, there is a scarcity of research addressing the comprehensive layout of tourism and leisure facilities [15–23]. Secondly, while POI data has been extensively utilized in urban function and land use planning studies [24–30], most applications are at a macro level, such as district and county. There is a dearth of research focusing on the micro-perspective, particularly at the street level and below, where population data could provide valuable insights [31–36]. Lastly, in terms of research methods, qualitative analysis or traditional spatial superposition are mostly used in existing studies on urban facilities planning and layout, while the number of quantitative studies is rather few [37]. Based on these factors, this paper focuses on the main urban area of Beijing, examining the distribution characteristics of existing tourism and leisure facilities and employing machine learning techniques for a quantitative simulation of their optimal location layout.

### Planning and layout practice of tourism and leisure facilities

Tourism and leisure facilities are fundamental to tourism and leisure activities, which do not only provide services to tourists, but also carriers of local culture and social development level. Tourism and leisure facilities can be defined as facilities that satisfy tourists’ sightseeing and leisure needs. These facilities usually contain World Heritage Sites, Tourist Attractions, Memorial Halls, Temples, City Squares, Parks, Aquariums, Botanical Gardens, Zoos, Resorts, etc. Other than provide diverse experience for tourists, tourism and leisure facilities are also key to fulfill local residents’ relaxation and social needs. Lin et al. proposed that the formation mechanism of the spatial agglomeration pattern of different types of leisure tourism resources is quite different, and the study provides a new idea for the analysis of tourism resources based on spatial big data [38]. In the context of metropolitan areas in China, such as Beijing, Shanghai, and Guangzhou, which are characterized by high population densities, the scholarly focus has been intensified. Meng’s investigation into the urban core’s transportation infrastructure reveals a progressive enhancement in the accessibility of leisure facilities. This improvement is attributed to the ongoing development of transportation networks, which are crucial for connecting residents with leisure amenities and enhancing their overall accessibility [39]. Beijing, Shanghai, and Guangzhou are areas with relatively high population density in China which have attracted extensive attention from relevant scholars. Meng’s research found that the construction of transportation facilities has continuously improved the transportation accessibility of leisure facilities in urban core areas. The rational layout of transportation should pay more attention to matching the spatial distribution of leisure facilities and population, focusing on areas with emerging population growth and leisure facilities resources.

The abundant area promotes the coordinated layout of transportation facilities and leisure service facilities [39]. Huang et al. believe that the spatial layout and location of leisure facilities in urban commercial centers should focus on the influence of companies, transportation facilities, and commercial residences, so as to facilitate the development and improvement of urban leisure facilities and provide convenience for leisure consumers [40]. Chen’s study reveals a positive correlation between the economic prosperity and population density of Shanghai’s central urban area and the prevalence of leisure facilities. The research indicates that while the proximity of transit stops influences the spatial distribution of these amenities, this impact does not intensify with increasing distance. Notably, there is a pronounced association between the location of transportation infrastructure and the establishment of leisure facilities, with the catering sector showing the most significant impact [41]. Jia et al. examined the spatial arrangement of public leisure facilities in Urumqi, identifying distinctive pattern characteristics that define the urban leisure space [28]. Zhang’s research pointed out that Beijing’s leisure space presents a trend of coexistence of high concentration in the center and ‘island-like’ distribution in the periphery. There are significant differences in the location selection of leisure facilities in various industries, and the characteristics of agglomeration at different scales are also different; the degree of specialization of the leisure industry in the central urban area is lower than in the peripheral edge areas, and the rational layout of leisure facilities plays an important role in improving urban happiness and optimizing the overall spatial layout of the city [42]. Tan et al. conducted an in-depth analysis of the ecological patterns and hotspot features of leisure tourism within Chongqing’s central urban district. Their research revealed a distinct circular spatial structure for leisure tourism hotspots, with categorized service formats clustered within this circular area. The distribution characteristics, scale, and degree of agglomeration are obviously differentiated [43]. The distribution of tourism and leisure facilities should not only meet the functional requirements of the city, but also take into account the differences in space needs of specific groups of people.

### Research purpose

The Facility Location Problem (FLP) is a multifaceted theoretical framework that examines the strategic spatial arrangement and efficient allocation of resources within human activity systems. Scholars have predominantly focused their research on key areas such as logistics planning and the strategic placement of emergency facilities, aiming to enhance operational efficiency and response preparedness [44–47]. The research methods mainly include model construction, algorithm design and spatial decision support [48–50]. Currently, the integration of facility location research with regional tourism development remains an under-explored area within academic discourse. This paper aims to address this gap by examining the strategic placement of tourism facilities within Beijing’s main urban district, thereby contributing to the existing body of facility location studies. Point of Interest (POI) data, a burgeoning resource in urban analytics, has gained traction in the assessment of urban functions and land use strategies. However, the prevalent applications of POI data have been concentrated on macro-level analyses, typically surpassing the district and county levels, while few studies on population data at street level or below [24–36]. Many studies have also made meaningful attempts on the distribution of urban public facilities by using POI data [15,16].

The site selection of tourism and leisure facilities in this paper refers to the determination of the number and location of tourism and leisure facility nodes in Beijing, in order to devise a rational and well-organized layout plan by balancing three key criteria: minimizing the overall cost, enhancing the service level, and maximizing the social benefits. This process is predicated on the establishment of several foundational elements, including the selection of the subject matter, the delineation of the target region, the definition of the cost function, and the specification of relevant constraint conditions [51].

## Research area and research methods

### Research area

The research area of this paper is 6 main urban areas in Beijing, which are, Haidian District, Fengtai District, Dongcheng District, Xicheng District, Chaoyang District and Shijingshan District. The main urban area covers 1366 square kilometers, accounting for 8.3% of the total area of the city. The main urban area is the core functional area and urban function extended area of Beijing, and the permanent resident population accounts for 50.2% of the city’s population. The GDP of the main urban area accounts for 72.9% of the total GDP of the city [52]. The “Beijing Municipal Master Plan (2016–2035)” sets forth a goal to achieve full coverage of the “15-minute Community Service Circle” by 2035. This approach applies the “life circle” philosophy at the community planning level, focusing on improving various service functions within a 15-minute walkable range. The services to be optimized include education, culture, healthcare, elderly care, sports, as well as tourism and leisure facilities [53].

This paper specifically defines the “15-minute Community Life Circle” as an area within approximately a 1-kilometer radius that can be reached by walking from a residential community within 15 minutes. Consequently, the study employs a 1km by 1km grid system as the analytical framework for the main urban area of Beijing.

### General research ideas

**Fig 1:**
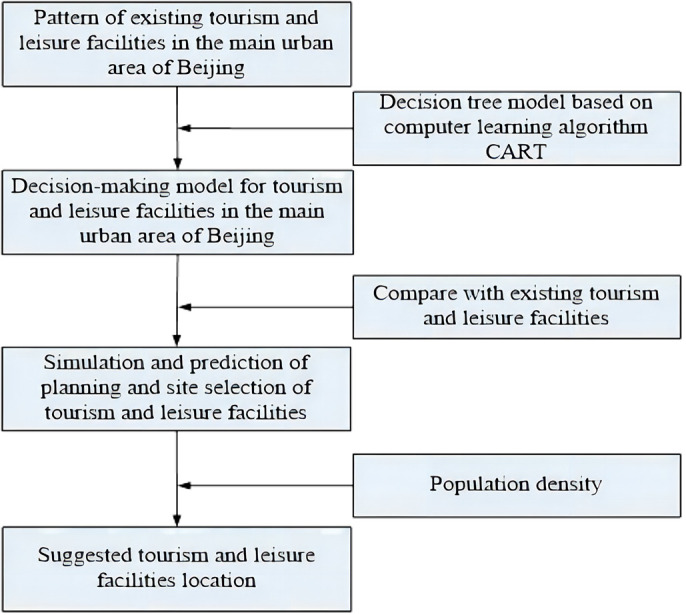
Research framework.

Fig 1 shows the research framework of the study. The pattern of current tourism and leisure facilities in main urban areas of Beijing was analyzed under the 1km × 1km grids map according to the 15 minutes walk service radius. According to the spatial distribution characteristics of the existing tourism and leisure facilities, machine learning algorithm (CART) was utilized to train the decision-making model for the site selection of tourism and leisure facilities. By comparing the simulation results of the model with the distribution of existing tourism and leisure facilities, the accuracy of the model can be determined. If the rate of AUC-ROC is more than 0.8, the model can be used to simulate and predict the suitability of a specific grid in the main urban area of Beijing for new tourism and leisure facilities. To further enhance the utility of the findings, it is imperative to consider the population density of each region, as this will facilitate the identification of priority locations for the development of tourism and leisure facilities.

### Research methods

There are many machine learning methods, and in the entity with both type and numerical variables, it is particularly important to choose the right algorithm. For example, support vector machine (SVM), a machine learning method based on statistical learning theory, VC dimension theory and structural risk minimization principle, has shown many unique advantages in solving small samples, nonlinear and high-dimensional pattern recognition problems [54]. However, the sample size needed to be processed in this paper is too large, and SVM cannot get good results. Another example is artificial neural network (ANN), which is a network structure that can be used to handle practical problems with multiple nodes and multiple output points [55]. This method has hardly been applied in the relevant fields of this paper, so this machine learning model is not considered in this paper.

Decision tree classification is a supervised learning method based on the known probability of occurrence of various situations and the size of the information entropy to obtain the probability that the maximum net present value is expected to be greater than 0 or equal to 0 [56]. Supervised learning is to give some known samples, each sample has a set of attributes, and the category to which the sample belongs is also known. According to the known attributes and categories of these samples, after training and learning, a sample of this type can be obtained. The classifier can then classify and judge new samples according to this classifier. In the entities with both type variables and numerical variables, it is particularly important to choose the appropriate algorithm.

Among decision tree algorithms, Classification and Regression Trees (CART) algorithm is a very effective non-parametric classification and regression method, which can deal with highly skewed or polymorphic numerical data, and can also deal with sequential or unordered generic data. First proposed by Breiman et al., it has been widely used in the field of statistics and data mining technology [57]. It constructs prediction criteria in a completely different way from traditional statistics. It is presented in the form of binary tree, which is easy to understand, use and explain. In many cases, the prediction tree constructed by CART model is more accurate than the algebraic prediction criteria constructed by common statistical methods, and the more complex the data and the more variables, the more obvious the superiority of the algorithm [25]. When CART is used as classification tree, its function is to analyze the characteristics of the research object to predict the category to which the object belongs [26], and the classification tree uses the GINI value as the basis for node splitting. When CRAT is used as a regression tree, the function is to predict the attributes of the object through the information of the object and represent it as a numerical value, and use the sample variance to measure the node purity. The higher the node purity, the better the node classification or prediction effect is.

### Generation of regression trees

According to the idea of data mining technology, the practical data is cleaned, selected and integrated to form a group of unknown and high purity data set, which is imported into the data warehouse. Let X_1_, X_2_, X_3_... X_n_ represent N attributes contained in a certain sample of the data, and use Y to represent the category to which the attributes belong. There is a fixed output value C_n_ on each attribute X_n_, and the regression tree model is expressed as:


$$\int \left(x\right)=\overset{N}{\underset{N=1}{\sum }}Cnl\left(x\epsilon Xn\right)$$ (1)

Select the jth attribute X_j_ and the value z of the attribute X_j_ in the data set as the segmentation point of the regression tree, this point can divide the data set into two areas: X_1_ = (j,z) = {x | x(j) ≤ z}X_2_ = (j,z) = {x | x(j) > z} Finding the best split point X_j_ is the point where the minimum squared difference is calculated:


$$\underset{j,s}{\min}\left[\underset{c_{1}}{\min}\underset{x_{1}\in R_{1}\left(j,s\right)}{\sum }{\left(y_{1}-c_{1}\right)}^{2}+\underset{c_{2}}{\min}\underset{x_{1}\in R_{2}\left(j,s\right)}{\sum }{\left(y_{1}-c_{2}\right)}^{2}\right]$$ (2)

### Classification tree generation

The CART algorithm recursively divides the N-dimensional space boundary of the dataset into non-overlapping rectangles, selects the best feature attribute by calculating the Gini index, and determines the best binary segmentation point of the attribute through the Gini index. The Gini index Gini(D) represents the uncertainty of the data set. The larger the Gini index of the attribute, the greater the uncertainty of the sample set.

Assuming that the probability that a point in the data set D belongs to the kth class is Pk, the Gini index formula of the probability distribution is:


$$Gini\left(p\right)=\sum_{K=1}^{K}p_{K}\left(1-p_{K}\right)=1-\sum_{K=1}^{K}{p_{K}}^{2}$$ (3)

Determine the existence of a certain point in attribute A in the data set D. Divide the data set D into two regions, D_1_ and D_2_, D_1_ = {(x,y) ∈ D | A(x) = a}, D_2_ = D and D_1_ in the attribute Under the fixed attribute of A, the Gini index of data set D is:


$$Gini\left(D,A\right)=\frac{D_{1}}{D}Gini\left(D_{1}\right)+\frac{D_{2}}{D}Gini\left(D_{2}\right)$$ (4)

### The specific process of CART classification tree establishment algorithm

**Step 1: Feature selection**

Select the feature with the highest information gain as the test feature, and use this feature to divide the node samples into subsets, which will make the mixing degree of different types of samples in each subset the lowest, and the information entropy required to divide the samples in each subset will be the least.

Information gain calculation


$$Info\left(D\right)=-\sum_{i=1}^{m}p_{i}\log_{2}p_{i}$$ (5)

$$Info_{A}\left(D\right)=\sum_{j=1}^{v}\frac{\left|D_{j}\right|}{\left|D\right|}\times Info\left(D_{j}\right)$$ (6)

$$Gain\left(A\right)=Info\left(D\right)-Info_{A}\left(D\right)$$ (7)

**Step 2: Generation of decision tree**

(1) Calculate the Gini index of all attribute features for the dataset. For each feature A, the attribute value of an existing tourism and leisure facility within grid is recorded as ‘1’, and the value without a tourism and leisure facility is recorded as ‘0’. This pair of features A can divide the dataset D into two regions, D1 and D2.
(2) For all features A and all feature segmentation points a in the data set, the feature with the smallest Gini index and the corresponding segmentation point are selected as the optimal feature and the best segmentation point.
(3) Do recursion (1)(2) on the current optimal subtree until the stopping condition is satisfied. The stopping condition of the recursive operation is that all samples in a node are of the same category, no features can be used to divide the node samples, or no samples can satisfy the value of the remaining features.
(4) Generate a CART classification tree.

**Step 3: Decision tree pruning**

The binary tree generated by the above algorithm has a large scale. Although the error rate obtained by learning set test is small, its true/error rate may be relatively large. A binary tree with low true error rate must be constructed by pruning technique. Overfitting often occurs when using decision trees for data mining. Pruning is the main method to solve the overfitting phenomenon in the decision tree learning process. This method mainly processes the generated decision tree and prunes the decision tree. In some subtrees or leaf nodes, the root node or parent node is used as a new leaf node to achieve the effect of simplifying the classification tree model and improving the classification speed and classification accuracy of the entire decision tree.

Decision tree pruning is a critical technique aimed at optimizing the model’s performance and generalizability, which can be conducted through two primary approaches: pre-pruning and post-pruning. Pre-pruning involves applying statistical criteria, such as information gain, prior to the construction of the tree to assess the viability of potential branches. A predefined threshold dictates whether a node should be split; if the subset’s statistical properties fall short of this threshold, the node remains unbranched. Striking the correct balance for the threshold is essential, as setting it too low can lead to an overly complex tree with excessive branches, complicating the interpretation of the resulting rules. Conversely, an excessively high threshold may result in a sparse tree with insufficient branches, potentially omitting crucial data and undermining the model’s predictive accuracy [58].

Post-pruning is the process of pruning off redundant branches after the decision tree is generated. Let the number of leaf nodes of the decision tree T be | T | . There are N_t_ sample points on the leaf node t, of which the number of sample points of class k is N_kt_, and H(T) is the empirical entropy on the node t. a>=0 is a parameter, so the loss function is defined as:


$$Ca\left(Tt\right)=C\left(Tt\right)+a\left|Tt\right|$$ (8)

C(T) represents the prediction error of the training data, | T | represents the complexity of the model, and the loss function actually expresses the balance between the two. The specific process is to calculate the empirical entropy T_a_ of each node, recursively traverse each node from the leaf node upwards, and determine whether the value of the loss function decreases after deleting a leaf node. If it decreases, the parent node is used as a new leaf node. Traverse all nodes until all nodes are judged.

## Data source and processing

### POI data

Data used in this study was captured from AutoNavi map on October 22–25, 2020 by GeoSharp1.0. The longitude and latitude coordinate system obtained by Amap is the projection coordinate system, and the conversion code is written by Python to convert its coordinates to the unified WGS84 geographical coordinate system.

### Population data

The population data used in this paper is the 100 m resolution grid population data set provided by WorldPOP project. This dataset is based on population data, assisted by night light remote sensing, land use (GeoCover 30 m resolution land cover dataset), distance factors to various land use types, elevation and slope information (calculated based on HydroSheds dataset) and other data, and calculated by random forest model and zoning density. It not only describes the overall characteristics of all residents, but also divides the population of different genders and age stages, based on which the population grid data of different groups is made. The data in the dataset is 1km raster data. This paper uses the data set of the main urban area of Beijing in 2020.

**Fig 2:**
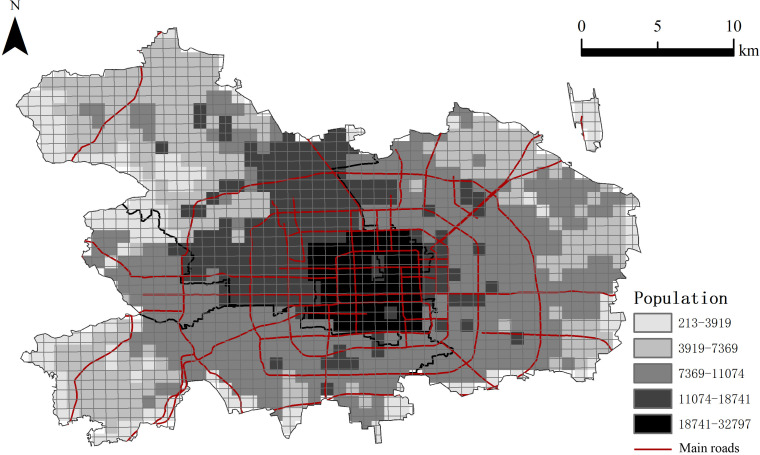
Distribution of population density in the main urban area of Beijing. This map was vectorized based on the standard map with the review number of Jing S (2022) 019, accessed October 22-25, 2020, at https://beijing.tianditu.gov.cn/bzdt/.

The resident population in the central urban area of Beijing is 10.988 million, accounting for 50.2% of the city’s total population. Within this demographic, the core area is home to 1.815 million residents, which constitutes 8.3% of the city’s population. Transportation infrastructure is one of the factors influencing the spatial distribution of population. As depicted in Fig 2, public service resources in Beijing are distributed in a gradient that diminishes from the city center outwards. This distribution pattern results in a significantly higher population density within the Third Ring Road, which mainly covers the areas of Dongcheng and Xicheng District compared to the outlying suburban regions. The inner part of Haidian District (the northwest part near the center) also has a higher population density than elsewhere. The population of residents in Beijing is also distributed along the subway lines, and the population in the Fifth Ring Road is basically distributed along several major subway lines such as Line 1, Line 2, Line 5, Line 10 and Line 13.

### Infrastructure classification and gridding

**Fig 3:**
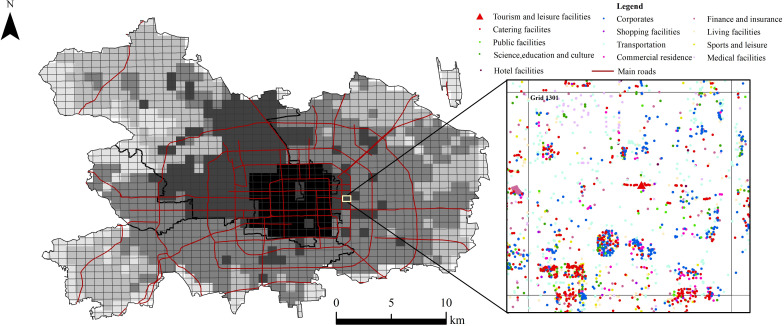
Grid division and partial schematic diagram of facilities in the main urban area of Beijing. This map was vectorized based on the standard map with the review number of Jing S (2022) 019, accessed October 22-25, 2020, at https://beijing.tianditu.gov.cn/bzdt/.

First, according to the relationship with tourism and leisure facilities, various types of urban facilities represented by POI are classified. It is mainly divided into 4 first-level categories, including basic service facilities, commercial service facilities, administrative facilities and leisure facilities. Transportation facilities, science, education and culture, medical facilities, public facilities, living facilities, finance and insurance, hotels, shopping, catering, government agencies and social groups, corporates, commercial residences, sports and leisure facilities, tourism and leisure facilities are 14 second-level categories, totaling 566,932 POI points (see Table 1). Then, taking a 15-minute walking distance as reference, and taking 1 km × 1 km as the scale unit, the main urban area of Beijing is divided into 1548 grids. Each grid has a unique number, which is convenient for exploring the spatial distribution of various facilities. Fig 3 shows an example of Grid No.1301, with one tourism and leisure facility in the grid and several other facilities displayed in different colors. After screening all grids with the spatial distribution of the various types of facilities, it is possible to obtain whether a certain type of facility is owned within each grid.

**Table 1:** Classification of POI data related to tourism and leisure facilities in the main urban area of Beijing.

Class I	Class II	Facility Name	Number of POIs (pieces)
Basic Service Facilities	Transportation Facilities	Airport, Railway Station, Long-Distance Bus Station, Subway Station, Bus Station, etc.	72247
	Science, Education and Culture	Kindergartens, Primary Schools, Middle Schools, Colleges and Universities, Driving Schools, Cultural Palaces, Training Institutions, Scientific Research Institutions, etc.	58500
	Medical Facilities	General Hospitals, Specialized Hospitals, Clinics, Pharmacies, Emergency Centers, Disease Control Centers, etc.	23278
	Public Facilities	Public Toilet, etc.	15717
	Living Facilities	Post Offices, Laundries, Maintenance Sites, Beauty Salons, Business Offices, etc.	44040
	Finance and Insurance	Banks, ATMs, Insurance Companies, etc.	21295
Commercial Service Facilities	Hotels	Hotels, Guesthouses, Express Hotels, Star-Rated Hotel, Apartments, etc.	4568
	Shopping	Supermarkets, General Shopping Malls, Shopping Centers, Digital Appliances, Markets, etc.	7550
	Catering	Chinese Restaurants, Fast Food Restaurants, Coffee Shops, Foreign Restaurants, Cafes, etc.	71008
Administrative Facilities	Government Agencies and Social Groups	Governments At All Levels, Social Groups, Industry Associations, Administrative Units, Welfare Institutions, etc.	46016
	Corporates	Companies, Cooperatives, Agriculture, Forestry and Horticulture Bases, etc.	132613
	Commercial Residences	Residential Areas, Industrial Parks, Dormitories, Villas, etc.	31455
Leisure Facilities	Sports and Leisure Facilities	Stadiums, Sports Venues, Playgrounds, Sports and Leisure Places, Agritainment, Cinemas, Sanatoriums, Picking Gardens, Leisure Places, etc.	31279
	Tourism and Leisure Facilities	World Heritage Sites, Tourist Attractions, Memorial Halls, Temples, City Squares, Parks, Aquariums, Botanical Gardens, Zoos, Resorts, etc	7366
Total			566932

## Decision on location of urban tourism and leisure facilities

According to the spatial distribution characteristics of the existing tourism and leisure facilities, a decision tree model under the CART algorithm is used to train a decision-making model for the location of tourism and leisure facilities in the main urban area of Beijing. Then, the model is used to simulate and predict each grid to see if it is suitable to build new tourism and leisure facilities.

### Establishment of decision tree for location selection of tourism and leisure facilities

First, match the grid with the classification of facilities. The criteria for whether a grid is suitable for tourism and leisure facilities is set as: if there are tourism and leisure facilities in a grid, it indicates that the objective environment of the grid is suitable for the site selection requirements of tourism and leisure facilities, which is classified as a positive class. On the other hand, if there are no tourism and leisure facilities in a grid, it is considered that the objective environment of the grid is not suitable for the site selection requirements of tourism and leisure facilities, and it is classified as a negative category. Then, the other types of facilities in the grid are also converted into binary classification: if there is a certain type of facility, the facility in the grid is a positive class, otherwise it is a negative class.

**Fig 4:**
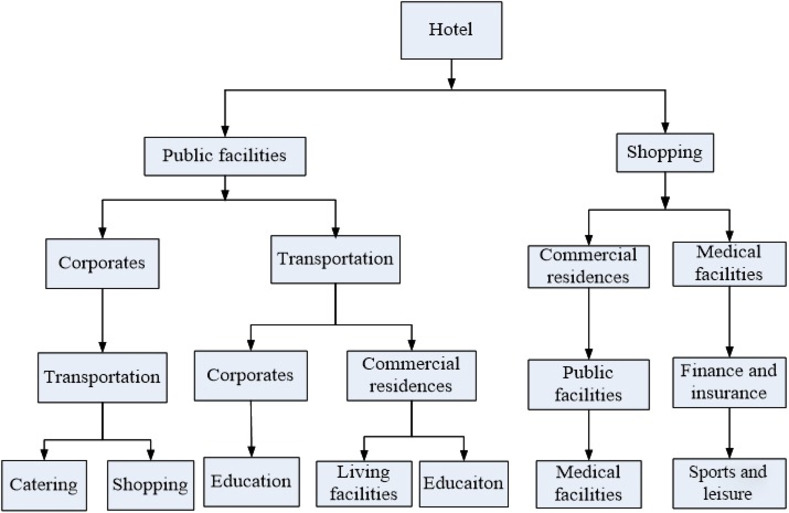
Decision-making model for location selection of tourism and leisure facilities.

The site selection for tourism and leisure facilities entails a dynamic decision-making process that iteratively evaluates the suitability of alternative plots based on the requirements of their surrounding areas, effectively filtering out those that fail to meet the established criteria. This approach aligns seamlessly with the decision tree algorithm’s methodology. In this process, the presence or absence of each type of supporting facility at a potential site represents a decision node within the tree, that leads to either inclusion or exclusion based on whether the site meets the necessary decision criteria. Employing the CART algorithm, the decision tree model is trained and refined using the existing facilities across all grids in Beijing’s main urban area as independent variables. The dependent variable is the presence or absence of tourism and leisure facilities. Through this training and learning process, a decision tree model is derived, which encapsulates the decision-making logic for site selection (as illustrated in Fig 4).

It can be seen from the results in Fig 4 that the first five layers of the decision tree contain 12 out of 13 types of functional facilities other than tourism and leisure facilities. Compared to other factors, government agencies and social groups are not as important during the process of site selection for tourism and leisure facilities, so it did not appear in the decision tree. The attributes in the upper layer of the decision tree are more important than the lower layer in the decision-making process. The model begins with hotels on top layer, emphasizing the dependency of tourism and leisure facilities on proximity to hotels, which also indicate hotel as the most significant influencing factor on location decisions.

The left side of the second layer indicates that tourism and leisure facilities would be built near public facilities if there is no hotel in the grid. The right side, on the other hand, shows that if tourism and leisure facilities are found in the same grid with hotels, it is highly possible that shopping amenities are near.

Moving to the left side of the third layer, the model acknowledges the significance of developed traffic when public facilities cannot be found nearby. If tourists cannot get access to public transportation, the utilization of tourism and leisure facilities would be affected. Simultaneously, the presence of commercial residences and medical facilities on the right side, shows the essential need for a well-rounded service ecosystem to a pleasant visit.

Financial facilities on the fourth layer indicates that tourists are also consumers, and they could use finance and insurance service during their tour. Together with services provided through public facilities, medical facilities, catering facilities and living facilities (the fifth layer), they could enjoy their trip to the full extent.

### Prediction of site selection for tourism and leisure facilities

**Fig 5:**
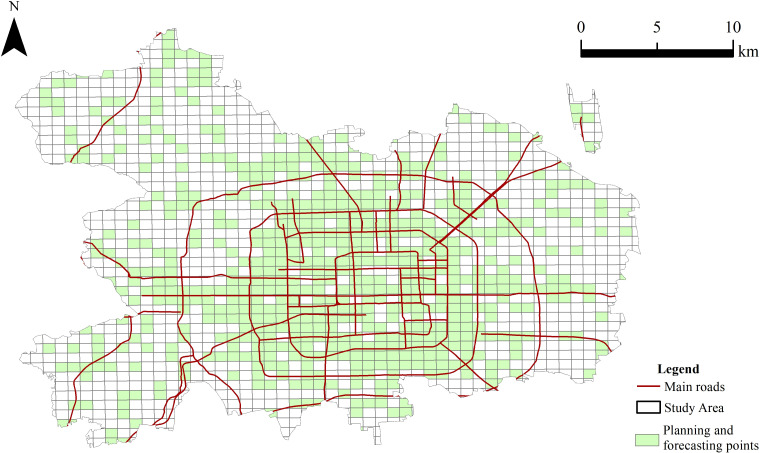
Preliminary prediction results of planning and layout of tourism and leisure facilities in the main urban area of Beijing. This map was vectorized based on the standard map with the review number of Jing S (2022) 019, accessed October 22-25, 2020, at https://beijing.tianditu.gov.cn/bzdt/.

By random sampling with equal positive and negative sample ratios performed from 1548 valid grids, 162 grids are finally screened out as training samples. Taking tourism and leisure facilities in each grid as the dependent variable and the other 13 types of facilities as independent variables, the decision tree prediction model was trained to predict the site selection suitability of 1548 grids in the main urban area of Beijing, and 629 grids were identified to be suitable for the construction of tourism and leisure facilities, shown as green grids in Fig 5. They are the forecasted points and the results of the decision tree model trained previously. In order to ascertain the model’s accuracy, the existing tourism and leisure facilities are utilized as a test set. The results demonstrate that 83.5% of the existing tourism and leisure facilities are consistent with the predicted results, thereby indicating the validity of the predicted results. Consequently, the model is worthy of further optimization.

The preliminary predictive analysis presented in Fig 5 also reveals distinct patterns in the distribution of existing tourism and leisure facilities within Beijing’s main urban area. A significant majority of these facilities are found within the grids that the model has predicted as suitable, corroborating the model’s accuracy. However, the analysis also identifies several predicted grids that, despite being conducive to the development of such facilities, are currently underserved or devoid of tourism and leisure infrastructure. For example, some grids in Shijingshan and Chaoyang district, which are situated away from the city’s central arteries, also meet the criteria for potential site development. This observation suggests that while the model’s predictions are largely aligned with the current distribution, it also uncovers opportunities for facility expansion in areas that are not optimally tapped.

### Re-filter based on population density

Despite the fact that the preliminary fitting of 629 sites has achieved the anticipated effect in predicting the selection of tourism and leisure facilities locations, the preliminary site selection outcomes are subjected to a re-screening process based on the degree of population density. This is intended to identify sites with high projected demand for construction in the near future, thereby enabling the government to accord these sites priority consideration in decision-making processes.

**Fig 6:**
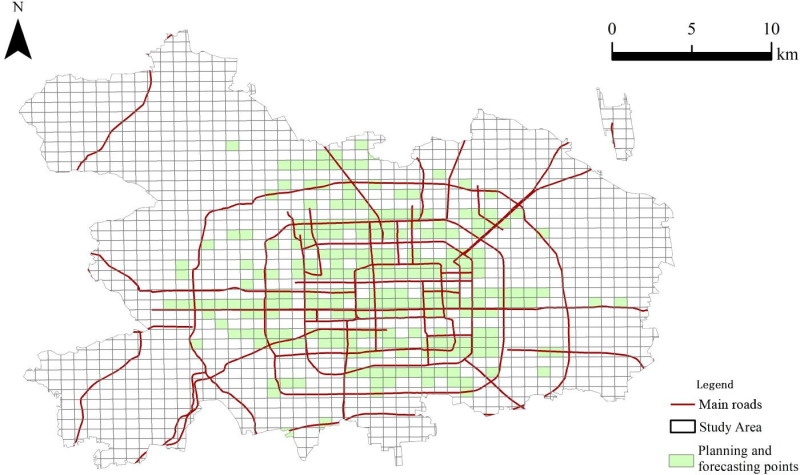
Final results of site selection for the planning and layout of tourism and leisure facilities in the main urban area of Beijing. This map was vectorized based on the standard map with the review number of Jing S (2022) 019, accessed October 22-25, 2020, at https://beijing.tianditu.gov.cn/bzdt/.

The selection of streets with a population density of more than 10,000 people was based on the available population data. These streets were identified as areas that should be prioritized for the construction of tourism and leisure facilities. The final results of the study revealed that there are 102 street areas in Beijing with a population density of more than 10,000 people, involving 373 grids. Subsequently, ArcGIS was employed to identify suitable sites within a 2 km radius of these 102 streets. This involved the removal of the existing layout of tourism and leisure facilities, resulting in the identification of 240 grids that were deemed suitable for priority sites. The specific distribution of these grids is illustrated in Fig 6. Within the main urban districts of Beijing, regions with significant population concentrations exhibit a notable clustering of existing tourism and leisure facilities. Despite this concentration, the predictive model indicates a substantial number of additional sites that are well-suited for future facility planning, underscoring the potential for expanded tourism and leisure infrastructure to meet the needs of the dense urban populace.

## Results and discussion

Based on POI big data and the population data, this study uses machine learning algorithm (CART algorithm) to simulate the location and layout of tourism and leisure facilities in the main urban area of Beijing. The research results show that:

(1)Utilizing the decision tree model derived from the CART algorithm, this study successfully developed a location decision model tailored for the siting of tourism and leisure facilities within Beijing’s primary urban district. The model’s predictive outcomes indicate a propensity for situating such facilities in proximity to hotels, underscoring the significant dependency of tourism and leisure infrastructure on accessible hospitality services. Additionally, the model suggests that the vicinity of public amenities and shopping centers serves as a secondary strategic location choice. Furthermore, the model emphasizes the importance of well-developed transportation networks in the site selection process, highlighting the need for tourism and leisure facilities to be conveniently accessible to enhance visitor experience and facility utilization. To sum up, accommodation availability and the quality of public and transportation infrastructure exert a considerable influence on the optimal location decision for tourism and leisure facilities.

(2) This study targeted the analysis of 1,548 valid spatial grids within Beijing’s primary urban area, employing a machine learning algorithm to develop a predictive model for the site selection of tourism and leisure facilities. The initial simulation yielded 629 potential locations deemed suitable for such facilities. To validate the model’s accuracy, it was cross-referenced with the existing tourism and leisure facilities, revealing a remarkable fit at a rate of 83.5%. This high degree of congruence attests to the model’s robustness and the method’s practical viability.

The spatial distribution of Beijing’s tourism and leisure facilities across the city exhibits a ‘point-like agglomeration and planar dispersion’ pattern. This configuration reflects a centralized layout strategy that aligns with the city’s central axis, characterized by a higher concentration of facilities in the central urban regions, in contrast to the more dispersed and fewer options in the peripheral areas.

(3) It is further identified 240 optimal sites for the strategic planning and layout of tourism and leisure facilities with demographic consideration. These sites are predominantly located across key urban districts of Beijing, including Dongcheng, Xicheng, Chaoyang, Haidian, Fengtai, and Shijingshan. While the current assessment indicates that certain grid areas within these districts are devoid of existing facilities, the study’s predictive model suggests that these areas present viable opportunities for future development. Notably, even districts situated away from the central thoroughfares, such as Shijingshan and parts of Chaoyang, have been found to contain numerous grids that are well-suited for the establishment of tourism and leisure infrastructure. Given the potential these areas hold for enhancing the city’s tourism and leisure landscape, it is recommended that authorities prioritize the development of a select number of these sites in the near term.

(4) Point of Interest (POI) data, renowned for its rich content, comprehensive coverage, and up-to-date relevance, serves as an invaluable asset in the realms of urban development and spatial layout optimization. This study pioneers the employment of machine learning algorithms, grounded in spatial data mining techniques, to enhance the planning and site selection processes for tourism and leisure facilities. By leveraging these algorithms, the study endeavors to achieve a globally optimal distribution of facilities, thereby minimizing the influence of planner subjectivity and fostering a more objective, scientific, and holistic approach to site selection.

The methodology applied in this study is not only broadly applicable across various urban planning scenarios but also holds significant reference value for the strategic location selection of a diverse array of public facilities. This underscores the potential for similar data-driven approaches to improve decision-making processes in urban planning and public service provision.

## Conclusion and future research

Site selection is a land-based choice, which makes location-based analysis and decision-making a necessary path. In the context of smart tourism city construction, site selection has emerged as a critical component, underpinning the strategic development of urban spaces that are both visitor-centric and technologically integrated [59–61]. Within the domain of smart tourism, the four key stakeholders—namely, public management and service sectors, tourism enterprises, local residents and tourists—each possess distinct entitlements and obligations that shape the smart tourism ecosystem and are beneficial for scientific site selection.

In the pursuit of smart tourism city management, city administrators are tasked with developing sophisticated models grounded in extensive location-based datasets. These models, enhanced by machine learning algorithms, serve as the foundation for delivering precise, data-driven intelligent services that are essential for the strategic development of tourism infrastructure. Such technological integration is pivotal for augmenting operational efficiency and the analytical prowess of decision-making processes [62–64]. When applied to the context of Beijing, this approach involves integrating the spatial arrangement of urban tourism and leisure facilities within a comprehensive public service framework. The establishment of tiered evaluation criteria enables a scientific management of these facilities, ensuring an optimized distribution that aligns with the leisure and tourism needs of residents. The utilization of urban Point of Interest (POI) data, enriched with location-specific information, offers a significant advantage by reducing the labor and time expenditure associated with traditional planning methods. It also mitigates the influence of planner subjectivity in site selection, thereby enhancing the objectivity and effectiveness of urban construction and spatial layout optimization [65–68].

Subsequently, it is imperative that tourism enterprises actively engage with and align their operations with the overarching strategies formulated by government-led public management and service sectors. Compliance with governmental directives and decisions is essential to ensure cohesive policy implementation and effective action. This collaborative approach is crucial for the strategic optimization of tourism and leisure facilities, ensuring that they are not only well-integrated into their local contexts but also contribute to creating environments that are inviting, accessible, and conducive to a high quality of life for residents and visitors alike.

Furthermore, the government should initiate the development of a diverse array of tourism and leisure facilities that cater to the everyday needs of the populace. This approach should prioritize foundational and essential services that enhance the quality of life for residents. There is also a need to innovate and reform the operational mechanisms governing these facilities. Tailored strategies should be devised according to the unique operational requirements of urban tourism and leisure layouts, with the aim of establishing a robust, multifaceted, and scientifically sound operational support system.

Lastly, the promotion of a healthy lifestyle is crucial to make the use of tourism and leisure facilities to the fullest. Employing diverse methods, efforts should be directed towards fostering and nurturing a sense of participation and habitual engagement among residents and tourists, thereby cultivating a society that actively partakes in and benefits from these amenities.

To conclude, it is essential to emphasize that the strategic placement of tourism and leisure facilities must consider the demographic distribution of local residents as a fundamental criterion. This selection should align with the city’s overarching planning and layout, ensuring integration with adjacent basic service facilities and harmonization with the environmental and cultural context of the scenic areas. The goal is to seamlessly blend these facilities into the natural and cultural landscapes, transforming them into integral components of the local scenery.

There are certain constraints of the study. While the findings offer a general framework for the site selection of tourism and leisure facilities, they do not address the nuanced site selection criteria for varied facility types. This represents an opportunity for future research to delve deeper into these distinctions.

From a pragmatic standpoint, future work should adopt a user-centric and application-oriented approach. Leveraging cutting-edge technologies such as big data analytics, the Internet of Things (IoT), and Generative AI, there is potential to significantly enhance the site selection process for tourism and leisure facilities [69–72]. By integrating these technologies into broader frameworks of urban planning, land resource management, and urban development, a more dynamic, harmonious, and sustainable system for urban tourism and leisure facilities planning can be established.

Our current research trajectory contemplates employing more sophisticated and adaptive methodologies for site selection, with a strong emphasis on intelligent applications. This will not only refine the strategic placement of facilities but also contribute to the evolution of a responsive and forward-thinking urban environment.
